# A Convenient Antiseptic Under-Sheet in Child-Birth

**Published:** 1894-05

**Authors:** 


A Convenient Antiseptic Under-sheet in Child-birth.—Ordinary tarred paper, such as is used in building, makes a very efficient protection in child-birth, etc., as an antiseptic and waterproof under-sheet. It is to be slipped under the ordinary sheet and when its usefulness is over may be rolled up and burned, the tar aiding combustion. It is cheap and always obtainable. Rolled into a cornucopia, of greater or lesser size, and secured by eyelets, staples or stitching, it is a convenient receptacle for dressings and refuse in surgical or medical cases. A small one filled with absorbent cotton or plaster or antiseptic sawdust may serve with advantage as a spit-cup in various diseases. These, like the sheet, can be easily burned.—*Ex.*

